# Contextualising Maximal Fat Oxidation During Exercise: Determinants and Normative Values

**DOI:** 10.3389/fphys.2018.00599

**Published:** 2018-05-23

**Authors:** Ed Maunder, Daniel J. Plews, Andrew E. Kilding

**Affiliations:** Sports Performance Research Institute New Zealand, Auckland University of Technology, Auckland, New Zealand

**Keywords:** fat oxidation, exercise, normative values, running, cycling

## Abstract

Using a short-duration step protocol and continuous indirect calorimetry, whole-body rates of fat and carbohydrate oxidation can be estimated across a range of exercise workloads, along with the individual maximal rate of fat oxidation (MFO) and the exercise intensity at which MFO occurs (Fat_max_). These variables appear to have implications both in sport and health contexts. After discussion of the key determinants of MFO and Fat_max_ that must be considered during laboratory measurement, the present review sought to synthesize existing data in order to contextualize individually measured fat oxidation values. Data collected in homogenous cohorts on cycle ergometers after an overnight fast was synthesized to produce normative values in given subject populations. These normative values might be used to contextualize individual measurements and define research cohorts according their capacity for fat oxidation during exercise. Pertinent directions for future research were identified.

## Introduction

During prolonged exercise, carbohydrate and fat are the primary substrates oxidized to fuel energy metabolism (Romijn et al., 1993; van Loon et al., 2001). Humans predominantly store carbohydrates as glycogen in skeletal muscle (Bergström and Hultman, 1967; Bergström et al., 1967) and the liver (Nilsson, 1973; Nilsson et al., 1973), with modest quantities also found in the brain, kidneys, and adipose tissue (Biava et al., 1966; Rigden et al., 1990; Meyer et al., 2002; Oz et al., 2003), and ~4 g circulating in plasma as glucose (Wasserman, 2009). Human carbohydrate storage is finite, and typically amounts to <3,000 kcal (<740 g) (Gonzalez et al., 2016), ~80% of which is in skeletal muscle and ~10–15% in the liver (Jensen et al., 2011). In contrast, human fat energy storage is effectively unlimited in the context of exercise (Gonzalez et al., 2016). Indeed, given 1 g of fat provides ~9.75 kcal of energy (Jeukendrup and Wallis, 2005), it can be estimated that even very lean individuals of 70 kg and 10% body fat possess ~68,250 kcal (7,000 g) of endogenous fat energy.

Carbohydrate is the quantitatively most important metabolic substrate during prolonged exercise of moderate-to-high intensities (Romijn et al., 1993; van Loon et al., 2001), and skeletal muscle glycogen can become depleted to near-zero concentrations after exercise of sufficient length and intensity (Ahlborg et al., 1967; Bergström and Hultman, 1967; Bergström et al., 1967; Hermansen et al., 1967; Hultman, 1967; Hultman and Bergström, 1967). Depletion of endogenous carbohydrate is therefore thought to limit prolonged exercise capacity in temperate conditions, with preferential depletion of glycogen sequestered in the intramyofibrillar compartment specifically implicated in impaired skeletal muscle function (Marchand et al., 2007; Nielsen et al., 2009, 2011, 2014; Ørtenblad et al., 2011) in the “localisation hypothesis” (Ørtenblad et al., 2013; Ørtenblad and Nielsen, 2015). Briefly, depletion of intramyofibrillar glycogen has been associated with impaired fatigue resistance (Nielsen et al., 2009) and tetanic Ca^2+^ handling (Ørtenblad et al., 2011; Nielsen et al., 2014), suggesting a role for these stores in excitation-contraction coupling, and therefore a role of their depletion in muscle fatigue. Importantly, intramyofibrillar glycogen is depleted at a relatively fasted rate during exercise than intermyofibrillar or sub-sarcolemmal glycogen, resulting in even lower intramyofibrillar compared to whole-muscle glycogen concentrations at fatigue (Marchand et al., 2007; Nielsen et al., 2011), which may serve to explain why fatigue during prolonged exercise can occur before whole-muscle glycogen concentrations approach zero.

In contrast, human fat reserves are effectively unlimited in the context of exercise, and so identifying the determinants of, and enhancing, fat oxidation during exercise is a pertinent training and research goal in endurance sport. Indeed, fat oxidation capacity has been correlated with performance in Ironman triathlons, which are ultra-endurance events (>8 h) in which carbohydrate availability is likely limiting (Frandsen et al., 2017). Maximizing fat oxidation is also likely of interest in a military context given the possible extreme duration and accompanying metabolic demand of field activities, which is of particular relevance when the logistical challenges associated with the provision of exogenous nutrition during military tasks are considered (McCaig and Gooderson, 1986). Lastly, fat metabolism is of great relevance in a health setting, given the observed positive and negative relationships between 24-h fat oxidation and markers of metabolic health such as insulin sensitivity and weight gain (Zurlo et al., 1990; Robinson et al., 2015), and that the capacity for fat oxidation during exercise has been associated with insulin sensitivity, metabolic flexibility, and lower metabolic risk factors (Venables and Jeukendrup, 2008; Rosenkilde et al., 2010; Robinson et al., 2015).

## Exercise intensity and whole-body fat oxidation

Perhaps the most fundamental determinant of whole-body fat oxidation rate is exercise intensity. The relationship between exercise intensity and fat oxidation is generally parabolic; with fat oxidation initially increasing with exercise intensity before declining at high work rates (Romijn et al., 1993), although it should be acknowledged that this parabolic relationship is not always observed, particularly in untrained cohorts (Bergman and Brooks, 1999). Reductions in whole-body fat oxidation at high intensities are likely largely mediated by a reduction in delivery of fatty acids to skeletal muscle. Plasma non-esterified fatty acid (NEFA) rate of appearance is reduced at high exercise intensities despite unchanged rates of peripheral lipolysis (Romijn et al., 1993), and intravenous infusion to enhance plasma NEFA availability increases whole-body fat oxidation rates at high exercise intensities (Romijn et al., 1995). The reduction in plasma NEFA availability and delivery to skeletal muscle is likely mediated by exercise intensity-induced reductions in adipose tissue blood flow (Spriet, 2014), which might itself be mediated by exercise intensity-induced increases in plasma catecholamine concentrations (Romijn et al., 1993).

However, impaired mitochondrial fatty acid uptake might also contribute to the reduction in whole-body fat oxidation observed at high exercise intensities, given the observed reduction in mitochondrial uptake and oxidation of long-chain fatty acids with increasing exercise intensity (Sidossis et al., 1997). This may be explained by exercise intensity-induced reductions in free carnitine availability (van Loon et al., 2001) and/or acidosis-induced suppression of muscle carnitine palmitoyltransferase I (CPT-I) activity (Starritt et al., 2000). Carnitine is a substrate in the CPT-I-catalyzed reaction resulting in mitochondrial fatty acid uptake (Fritz and Yue, 1963), and the reduced pH (7.0–6.8) in the aforementioned study (Starritt et al., 2000) is physiologically reasonable during prolonged vigorous exercise (Sahlin et al., 1976). Therefore, the reduction in whole-body fat oxidation seen at high exercise intensities may be governed by reduced fatty acid delivery to *and* uptake in skeletal muscle.

### The “Fat_max_” test

**Figure 1:**
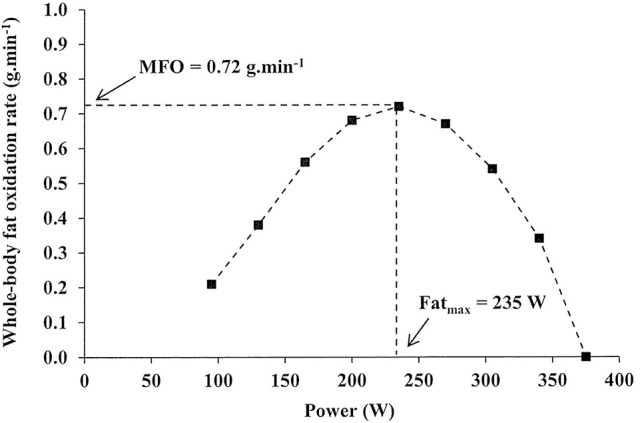
Representative illustration of fat oxidation (g.min^−1^) against exercise intensity (W) during a graded, cycling Fat_max_ test, where MFO, maximal rate of fat oxidation (g.min^−1^) and Fat_max_, the intensity at which MFO occurs (W).

In order to comprehensively define the relationship between whole-body fat oxidation rate and exercise intensity, the “Fat_max_” test was developed (Achten et al., 2002). This graded exercise test elucidates whole-body fat oxidation rates across a range of exercise intensities, the maximal rate of fat oxidation (MFO), and the intensity at which the MFO occurs (Fat_max_) using indirect calorimetry (Figure 1). This test advances on previous protocols using four incremental submaximal workloads (Pérez-Martin et al., 2001) that, for optimal use, require an initial assessment directly measuring maximal aerobic power (Gmada et al., 2012; Marzouki et al., 2014). The original “Fat_max_” protocol consisted of 5-min, 35-W step increments performed after an overnight fast on a cycle ergometer until the respiratory exchange ratio reached 1.0, after which 2-min 35-W steps were employed (Achten et al., 2002). Importantly, this study found no significant difference in Fat_max_ in a sub-set of well-trained participants asked to perform an additional 3-min step test, although it should be acknowledged that step durations of 6 min may be required for sedentary individuals to reach steady-state (Bordenave et al., 2007). Finally, participants were asked to perform continuous bouts of cycling (>35 min) at single exercise intensities corresponding to those on the Fat_max_ test, and differences in MFO or Fat_max_ were not significant in the first 5 min or when averaged over the course of these prolonged assessments compared to results in the 5-min step test. Thus, the authors concluded two key theoretical limitations of step-test determination of substrate metabolism, namely shifts in substrate utilization over time and effects of prior steps, were not influential (Achten et al., 2002). The 3-min step protocol described here is indicative of those used in the literature subsequently (Achten and Jeukendrup, 2003a,b, 2004), while the starting workload and work increment magnitude is adjusted in accordance with participant training status (Rosenkilde et al., 2010; Mora-Rodríguez et al., 2016; Dandanell et al., 2017a). Importantly, a sufficiently low starting workload may effectively obviate the requirement for a specific “warm-up” protocol. Conceptually identical treadmill protocols have been used (Achten et al., 2003), and some researchers have conducted assessments in the fed state (Stisen et al., 2006; Gonzalez-Haro et al., 2007; Schwindling et al., 2014). This relatively short protocol duration makes Fat_max_ testing a viable monitoring tool for endurance athletes concerned with substrate metabolism during competition. Lastly, the practicality of this protocol is particularly important given attempts to predict MFO and Fat_max_ based on heart rate, power, and estimated maximum oxygen uptake (VO_2max_) have not been successful (Brun et al., 2011).

The reliability of Fat_max_ assessments has been examined. The first reliability study of the Fat_max_ protocol described above reported a coefficient of variation (CV) of 9.6% for Fat_max_ in a cohort of overnight fasted moderately-trained males with 24-h pre-trial dietary repetition (Achten and Jeukendrup, 2003a). Interestingly, a similar study reported a CV of just 3% for Fat_max_ and 11% for MFO (Dandanell et al., 2017b). These CVs are similar to those for MFO measured in sedentary cohorts using 4–5 pre-defined submaximal workloads based on prior assessment of maximal aerobic power (Gmada et al., 2012; Marzouki et al., 2014). In contrast, a 6-min step test used to determine Fat_max_ in a heterogeneous cohort of healthy males and females demonstrated wide limits of agreement and therefore considerable intra-individual variability (Meyer et al., 2009). However, and critically, pre-trial diet and menstrual cycle was not controlled in this study, likely contributing to intra-individual variability given the reported influence of these variables on substrate oxidation during exercise (Arkinstall et al., 2001; Campbell et al., 2001). Indeed, reliability of a similar treadmill protocol with 24-h dietary control conducted after an overnight fast reported CVs of 7 and 5% for MFO (g.min^−1^) and treadmill velocity at MFO (km.h^−1^), respectively (De Souza Silveira et al., 2016). However, high CVs (>15%) have been reported with 24-h dietary control (Croci et al., 2014a). The reason for this disparity in reliability is unclear, but may be related to the *effectiveness* of the pre-exercise dietary and exercise control measures (Astorino and Schubert, 2017). Failing to adequately match pre-exercise muscle glycogen content is likely to impact MFO given muscle glycogen availability is an independent regulator of substrate metabolism during exercise (Hargreaves et al., 1995).

As described above, the validity of the original Fat_max_ protocol was examined against prolonged exercise bouts at intensities equivalent to those in the step test, with results from the step test demonstrated to be reflective of those over longer duration (Achten et al., 2002). Interestingly, Schwindling et al. (2014) had trained cyclists perform step Fat_max_ tests, and then 1-h constant-load tests at Fat_max_, one workload above Fat_max_, and one workload below Fat_max_. No significant differences in absolute fat oxidation rates were observed between-intensities in the 1-h bouts, suggesting that results from short-duration Fat_max_ tests may *not* be reflective prolonged exercise. Therefore, Fat_max_ testing might be used to quickly and non-invasively monitor metabolic adaptations to training, rather than to elucidate the metabolic consequences of given exercise bouts, which might require prolonged, steady-state assessments. Indeed, MFO has recently been correlated with performance in Ironman triathlon (*r* = 0.35, *P* < 0.01) (Frandsen et al., 2017), which supports its utility in training monitoring for endurance events likely limited by carbohydrate availability. Regarding the use of Fat_max_ assessments for deriving training prescriptions, statistical similarity has been observed between Fat_max_ and the intensity at which the first increase of plasma lactate concentration (LIAB) occurs (Achten and Jeukendrup, 2004; Tolfrey et al., 2010), whilst it appears Fat_max_ occurs at a greater relative intensity than the ventilatory threshold (Venables et al., 2005). Agreement between Fat_max_ and the lactate threshold has not always been observed, although it should be acknowledged that the dietary controls employed in this study were unclear (González-Haro, 2011).

In a health context, MFO has been significantly positively correlated with insulin sensitivity in a large cohort (*N* = 57) of young, healthy males (Robinson et al., 2015), and absolute Fat_max_ (Watts) has been positively correlated with insulin sensitivity in non-insulin-resistant obese males (Lambert et al., 2017). This link might be explained by mitochondrial function, given β-oxidation of fatty acids to acetyl CoA, oxidation of fatty acid or non-fatty acid-derived acetyl CoA in the citric acid cycle, and oxidative phosphorylation along the electron transport chain all occur in the mitochondria (McBride et al., 2006; Holloszy, 2011; Wu et al., 2014), and that increases in mitochondrial volume density (Hoppeler et al., 1985; Montero et al., 2015), mitochondrial oxidative capacity (Granata et al., 2016a,b), and mitochondrial enzyme content and activity (Spina et al., 1996; Scalzo et al., 2014; Granata et al., 2016a) occur in response to exercise training. Mechanistically, low mitochondrial activity may be linked to insulin resistance and development of type 2 diabetes via exacerbated production of reactive oxygen species and/or impaired lipolytic enzyme activity and accumulation of intracellular lipids, resulting in impaired regulation of insulin signaling and glucose transport (Wang et al., 2010). Indeed, mitochondrial fat oxidation capacity has been negatively correlated with whole-body respiratory exchange ratio during exercise (Sahlin et al., 2007), whilst training-induced increases in exercise-induced whole-body fat oxidation have been correlated with improvements in mitochondrial respiration and citrate synthase activity (Bordenave et al., 2008). Given the already well-established relationship between cardiorespiratory fitness and a range of metabolic and cardiovascular disease outcomes (Harber et al., 2017), and the American Heart Association's recent advocacy of cardiorespiratory fitness or maximum oxygen uptake (VO_2max_) testing in cardiovascular disease risk assessment (Ross et al., 2016), it is possible that quantifying MFO within these assessments will emerge as a tool to improve their predictive power. However, this would require longitudinal studies investigating associations between changes in MFO and metabolic risk factors such as insulin sensitivity.

Therefore, Fat_max_ tests appear a practical monitoring tool in performance settings where the capacity to utilize fat as a metabolic substrate is of concern, and might also be useful in clinical exercise physiology as an indicator of metabolic health. The purpose of the present review is to extend previous summaries (Jeukendrup and Wallis, 2005; Purdom et al., 2018) by systematically exploring key determinants of MFO and Fat_max_ for consideration during laboratory assessment, and to for the first time contextualize individually measured values in given subject populations with normative values. Normative values could be used to define the fat oxidation capacity of given research cohorts in exercise-metabolic studies in a manner analogous to VO_2max_-based definitions of aerobic capacity. Key directions for future research will be discussed.

## Maximal fat oxidation: what we know

In order to explore the determinants of MFO and Fat_max_, a systematic literature search was performed to identify all studies using Fat_max_ protocols in adult populations. As such, “maximal fat oxidation,” “peak fat oxidation,” and “Fat_max_” were searched in the PubMed and Web of Science databases (27/03/2018). Hand searches of reference lists and key journals were also conducted. Studies published in English and reporting directly measured MFO and/or Fat_max_ values in adult populations were included. This search approach yielded 53 studies for inclusion in the review.

### Training status

Five studies were identified that directly compared MFO and/or Fat_max_ between subjects groups of different training status (Nordby et al., 2006; Stisen et al., 2006; Lima-Silva et al., 2010; Schwindling et al., 2014; Ipavec-Levasseur et al., 2015). In comparisons of trained endurance athletes with different levels of VO_2max_, the better-trained group has greater MFO, with no difference in Fat_max_ (Lima-Silva et al., 2010; Schwindling et al., 2014). Those studies comparing active with untrained individuals have observed significantly greater MFO (Nordby et al., 2006; Ipavec-Levasseur et al., 2015), or a tendency toward greater MFO (Stisen et al., 2006), in the active or trained group, with only one of these studies reporting a difference in Fat_max_, which was greater in the trained group (Nordby et al., 2006). Alternatively, five large cohort studies with heterogeneous subject populations have all reported a significant small-to-moderate influence of VO_2max_ on MFO (Venables et al., 2005; Robinson et al., 2015; Fletcher et al., 2017; González et al., 2017; Randell et al., 2017).

A moderating effect of training status on MFO is not surprising given the previously observed significantly higher whole-body fat oxidation rates in trained compared to untrained males exercising at the same absolute workload (van Loon et al., 1999). Indeed, as a result of exercise training, skeletal muscle adaptations occur that augment fat oxidation during exercise (Egan and Zierath, 2013). These include mitochondrial biogenesis (Howald et al., 1985), increased tricarboxylic acid cycle enzyme and electron transport chain protein content (Egan et al., 2011), and increased fatty acid transporter and enzyme content (Talanian et al., 2010). An interesting direction for future research might be to compare MFO and Fat_max_ between trained endurance athletes competing in events with different requirements for fat oxidation, e.g. traditional endurance events such as half-marathon and marathon running and ultra-endurance events such as Ironman triathlons, and also to derive data from elite-level endurance populations.

### Sex

Seven studies were identified that compared males (*N* = 439) and females (*N* = 390) in terms of absolute MFO (g.min^−1^) and/or Fat_max_ (%VO_2max_) (Bircher et al., 2005; Venables et al., 2005; Bogdanis et al., 2008; Carey, 2009; Chenevière et al., 2011; Bagley et al., 2016; Fletcher et al., 2017). In order to quantitatively elucidate sex-mediated effects on these variables, sample size-weighted means and standard deviations (SD) for males and females were calculated. Standard error was converted to SD through multiplication by the square root of the sample size (Altman and Bland, 2005). SD for each study was collapsed by first squaring and then multiplying by the degrees of freedom. A sample size-weighted overall SD was calculated as the square root of the sum of collapsed SDs divided by total degrees of freedom. Cohen's *d* effect sizes (ES ± 90% confidence limits) were subsequently computed and interpreted according to Cohen's criteria (Cohen, 1977). Results from this analysis suggest absolute MFO is greater in males (*N* = 270, 0.56 ± 0.17 g.min^−1^) than females (*N* = 236, 0.44 ± 0.15 g.min^−1^), an effect of *large* magnitude (ES = 0.76 ± 0.10). However, Fat_max_ appears greater in females (*N* = 344, 56 ± 14%VO_2max_) than males (*N* = 371, 51 ± 14%VO_2max_), an effect of *small* magnitude (ES = 0.41 ± 0.09). These effects are aligned to those in a recent large-scale (*N* = 305; MFO, 0.62 ± 0.19 vs. 0.48 ± 0.15 g.min^−1^, *P* < 0.0001, ES = 0.76 ± 0.13; Fat_max_, 59 ± 16 vs. 62 ± 16% VO_2max_, *P* = 0.09, ES = 0.19 ± 0.13; in males and females, respectively) cohort study (Fletcher et al., 2017).

However, some studies making comparisons between-sexes have reported MFO relative to fat-free mass (FFM). When expressed in these terms (mg.kg FFM^−1^.min^−1^), two large cohort studies have reported greater MFO in females compared to males (Venables et al., 2005; Fletcher et al., 2017). This effect has been observed in moderately trained individuals (Chenevière et al., 2011), and a tendency toward this effect has been observed in a poorly-defined active cohort (Bagley et al., 2016). Interestingly, it appears this effect is abolished in overweight/obese individuals (Bogdanis et al., 2008; Haufe et al., 2010). In accordance with these findings, it has been observed that females have greater relative whole-body fat oxidation (i.e., as a percentage of overall energy expenditure) at given steady-state exercise intensities compared to males (Knechtle et al., 2004), indicative of greater reliance on fat metabolism during exercise in females. The ovarian hormone estrogen may explain this sex difference (Oosthuyse and Bosch, 2010; Devries, 2016), as estrogen appears to stimulate lipolysis and NEFA availability (D'Eon et al., 2002), plausibly via activation of 5′ adenosine monophosphate-activated protein kinase (AMPK) (D'Eon et al., 2005).

The existing literature therefore suggests that whilst absolute MFO is generally greater in males compared to females, MFO relative to FFM is likely greater in non-obese females compared to non-obese males. There also appears a minor tendency toward greater Fat_max_ in females compared to males. Given sex-related differences in body mass and composition, MFO relative to FFM might be more descriptive when comparing between sexes. Whether these effects are observed in endurance-trained cohorts is unknown. Similarly, effects of the menstrual cycle on MFO and Fat_max_ have not been studied, but warrant consideration in the context of serial inter-individual measurement.

### Nutritional status

Only one study has directly examined the effect of acute feeding status on MFO and Fat_max_ (Achten and Jeukendrup, 2003b). Trained males performed Fat_max_ assessments on a cycle ergometer after an overnight fast, with 75 g of glucose or placebo ingested 45 min pre-exercise. MFO (0.33 ± 0.06 vs. 0.46 ± 0.06 g.min^−1^) and Fat_max_ (52 ± 3 vs. 60 ± 2%VO_2max_) were significantly decreased with pre-exercise carbohydrate feeding (Achten and Jeukendrup, 2003b). This is likely explained by carbohydrate-induced insulinaemia, suppression of lipolysis, and suppression of fatty acid availability, which in turn might be expected to suppress whole-body fat oxidation in a manner similar to that seen at high exercise intensities (Romijn et al., 1995). Indeed, triglyceride and heparin infusion has been shown to increase plasma NEFA concentration, whole-body lipolysis, and fat oxidation rate during exercise with pre-exercise glucose feeding toward values observed during exercise after an overnight fast, suggesting that part of the suppressive effect of pre-exercise carbohydrate feeding on whole-body fat oxidation is explained by reduced fatty acid availability (Horowitz et al., 1997). Acute nutritional status is therefore a clear determinant of MFO and Fat_max_, and should be considered when comparing results between-studies as well as in serial intra-individual assessment. However, further examination of this effect in untrained populations is warranted, as is the time-course and macronutrient content of pre-exercise feeding on measures of MFO and Fat_max_. Such data might provide exercise physiologists with guidelines when using Fat_max_ tests for athlete monitoring and in health assessments, as conducting assessments at the exact same time of day is not always possible.

From a chronic dietary perspective, a recent large study of 150 male and 155 female subjects used hierarchical regression to elucidate the influence of a 4-day dietary record on MFO, and reported absolute carbohydrate and fat intakes accounted for 3.2% of the variation, with carbohydrate and fat intakes contributing negatively and positively to MFO, respectively (Fletcher et al., 2017). Whilst the degree of variance explained by diet was small in this mixed-cohort study, this contribution might be greater in homogenous cohorts. Nevertheless, an independent effect of chronic macronutrient intake was observed, making it therefore a critical variable to control in repeat testing.

In a cross-sectional study involving a homogenous cohort of male ultra-endurance runners, MFO (1.54 ± 0.18 vs. 0.67 ± 0.14 g.min^−1^) and Fat_max_ (70 ± 6 vs. 55 ± 8%VO_2max_) were significantly higher in those habitually consuming a ketogenic vs. high carbohydrate diet (Volek et al., 2016). Habitual consumption of a ketogenic diet was defined as a diet deriving < 20% of energy from carbohydrate and >60% from fat, whereas a high-carbohydrate diet was one that derived >55% of energy from carbohydrate, as confirmed by a 3-day weighed food record. A greater whole-body fat oxidation rate was observed during prolonged steady-state exercise in the low-carbohydrate group (~60%), an adaptation consistently seen in diet intervention studies (Phinney et al., 1983; Burke et al., 2000). Interestingly, however, muscle glycogen utilization during prolonged steady-state exercise was not significantly different between-groups, suggesting habitual consumption of a ketogenic diet did not spare glycogen in *working* skeletal muscle (Volek et al., 2016), which indicates the carbohydrate sparing effect was explained by reduced hepatic glycogenolysis and glucose output (Webster et al., 2016). An interesting direction for future research would be to determine the “threshold” of carbohydrate restriction required to elicit changes in MFO and Fat_max_, as this might provide endurance athletes with pertinent information when preparing events where maximizing fat utilization, and minimizing endogenous carbohydrate utilization, is sought. This might be particularly useful in a military context when long-duration tasks are performed (McCaig and Gooderson, 1986).

It is also possible that protein intake exerts an effect on MFO. During 3-month consumption of a weight-maintenance diet, increasing protein intake by ~10 g.d^−1^ has been shown to significantly increase MFO by ~19% in a mixed-sex sample of previously weight-stable volunteers (Soenen et al., 2010). Importantly, the increase in protein intake explained ~39% of the increase in MFO. These results implicate modifying protein consumption as a potential strategy to alter MFO, although the contribution of the inevitably reduced daily carbohydrate consumption on MFO in this study was not quantified.

### Exercise modality

A further consideration is exercise modality. In general, studies comparing running and cycling at given exercise intensities have reported greater fat and reduced carbohydrate oxidation rates during running (Snyder et al., 1993; Achten et al., 2003; Knechtle et al., 2004; Chenevière et al., 2010). However, comparisons of MFO and Fat_max_ between-modalities have not been as conclusive. The original study reported significantly greater MFO (0.65 ± 0.05 vs. 0.47 ± 0.05 g.min^−1^), with no difference in Fat_max_ (62 ± 3 vs. 59 ± 3%VO_2max_), during treadmill running compared to cycling in moderately-trained males (Achten et al., 2003). A further study in a similar subject population failed to observe a significant difference in MFO, but did observe a greater Fat_max_ during running (Chenevière et al., 2010). The reason for this disparate result in terms of MFO is not easily discernible, but could be related to between-study differences indirect calorimetry (analysis of 1 vs. 2 min of expired gases per 3-min stage), given the greater VO_2_ slow component during cycling (Billat et al., 1999). It is therefore recommended that the exercise modality in which Fat_max_ tests are performed be considered when between-study and intra-individual comparisons are made, and by those preparing for multi-modal endurance competitions such as triathlons.

### What we know: conclusions

It has been demonstrated that the training status, sex, and acute and chronic nutritional status of the subject population or individual under study are clear determinants of MFO and Fat_max_, with a possible effect of exercise modality. These determining factors must be considered when interpreting results between-studies and in serial intra-individual measurement.

## Maximal fat oxidation: normative values

Given the interest in measurement of MFO and Fat_max_ in research and non-research settings, it would be prudent to generate normative values from existing data in order to contextualize individually measured values and define the fat oxidation capacity of given research cohorts. However, in order to do this, the aforementioned determinants of MFO and Fat_max_ need to be considered. Accordingly, published MFO and Fat_max_ values were synthesized from studies with homogeneous cohorts performing assessments after an overnight fast on a cycle ergometer. These criteria were applied in order to generate sufficient data to produce meaningful normative values.

Studies were subsequently partitioned into five populations: endurance-trained, lean males (Achten et al., 2002, 2003; Achten and Jeukendrup, 2003a,b, 2004; Nordby et al., 2006; Frandsen et al., 2017), recreationally-active, lean males (Bircher et al., 2005; Croci et al., 2014a,b; Guadalupe-Grau et al., 2014; Lanzi et al., 2014; Bagley et al., 2016), recreationally-active, lean females (Bircher et al., 2005; Isacco et al., 2015; Bagley et al., 2016), overweight/obese males (Mogensen et al., 2009; Rosenkilde et al., 2010; Ara et al., 2011; Tsujimoto et al., 2012; Alkahtani et al., 2013; Alkahtani, 2014; Lanzi et al., 2014, 2015; Ipavec-Levasseur et al., 2015; Mohebbi et al., 2015; Nordby et al., 2015; Mora-Rodríguez et al., 2016; Dandanell et al., 2017b), and overweight/obese females (Besnier et al., 2015; Borel et al., 2015; Dandanell et al., 2017b). “Endurance-trained” was defined by a sample mean VO_2max_ >55 ml.kg^−1^.min^−1^ and active engagement in training for endurance events. “Recreationally-active” was defined as physically active according to the individual study, not training for endurance events, and, where measured, by a sample mean VO_2max_ <55 ml.kg^−1^.min^−1^. The division between “lean” and “overweight/obese” was defined in males as a body fat percentage of 25% and/or body mass index of 25 kg.m^−2^, and in females as a body fat percentage of 30% and/or body mass index of 25 kg.m^−2^. Owing to often-absent definitions of physical activity status in overweight populations, those considered overweight/obese were not further defined by physical activity status. Baseline values were used for intervention studies. For synthesis, a sample size-weighted mean and SD for MFO was calculated for each population as described above for sex-mediated comparisons (see section Sex). Subsequently, normative percentile values were generated for each population assuming a within-population normal distribution (Tables 1, 2).

**Table 1:** Normative percentile values for MFO (g.min^−1^) in different subject populations during assessments performed on a cycle ergometer after an overnight fast.

**Population**	***N***	**Mean MFO (g.min^−1^)**	**20th percentile**	**40th percentile**	**60th percentile**	**80th percentile**
Endurance-trained, lean males	201	0.53 ± 0.16	0.40	0.49	0.58	0.67
Recreationally-active, lean males	105	0.46 ± 0.14	0.34	0.42	0.49	0.58
Recreationally-active, lean females	68	0.35 ± 0.12	0.25	0.32	0.38	0.45
Overweight/obese males	193	0.28 ± 0.14	0.16	0.24	0.31	0.39
Overweight/obese females	144	0.16 ± 0.05	0.12	0.15	0.17	0.20

*For example, measurement of MFO at 0.67 g.min^−1^ in an endurance-trained, lean male would place them in the 80th percentile*.

**Table 2:** Normative percentile values for Fat_max_ (%VO_2max_) in different subject populations during assessments performed on a cycle ergometer after an overnight fast.

**Population**	***N***	**Mean Fat_max_ (%VO_2max_)**	**20th percentile**	**40th percentile**	**60th percentile**	**80th percentile**
Endurance-trained, lean males	201	56 ± 8	49	54	58	63
Recreationally-active, lean males	67	51 ± 8	44	48	53	58
Recreationally-active, lean females	38	50 ± 10	41	47	52	58
Overweight/obese males	190	43 ± 18	28	38	47	57
Overweight/obese females	27	61 ± 10	52	58	64	70

*For example, measurement of Fat_max_ at 63%VO_2max_ in an endurance-trained, lean male would place them in the 80th percentile*.

A trend toward greater MFO with increasing training status was observed (Table 1), and in males compared to females, which supports the evidence from individual studies presented above. Similarly, a less-pronounced trend toward greater Fat_max_ with increasing training status was observed (Table 2), with the exception of overweight/obese females, although this may be an artifact of the small sample size (*N* = 27). These normative percentile values might therefore be used by exercise physiologists to contextualize individual measurements and define the fat oxidation capacity of given research cohorts, whilst acknowledging the aforementioned determinants of MFO when making inferences. It is worth noting that no data was available for endurance-trained female populations, which is a pertinent area for future research. However, it might be possible to use the values reported for endurance-trained males and scale them down according to the synthesis described above for sex-mediated comparisons, which demonstrated MFO was on average 28% greater in males (0.56 ± 0.17 vs. 0.44 ± 0.15 g.min^−1^). It should also be noted that none of this data was derived from studies in which participants ingested a high-fat or ketogenic diet, which is known to increase fat oxidation during exercise (Phinney et al., 1983; Burke et al., 2000). Indeed, in many of the studies in endurance-trained males participants were specifically instructed to ingest a high-carbohydrate meal the evening before testing (Achten et al., 2002, 2003; Achten and Jeukendrup, 2003a,b, 2004). Therefore, these values are likely only of relevance to those ingesting a traditional mixed diet.

## Maximal fat oxidation: what we don't know

**Figure 2:**
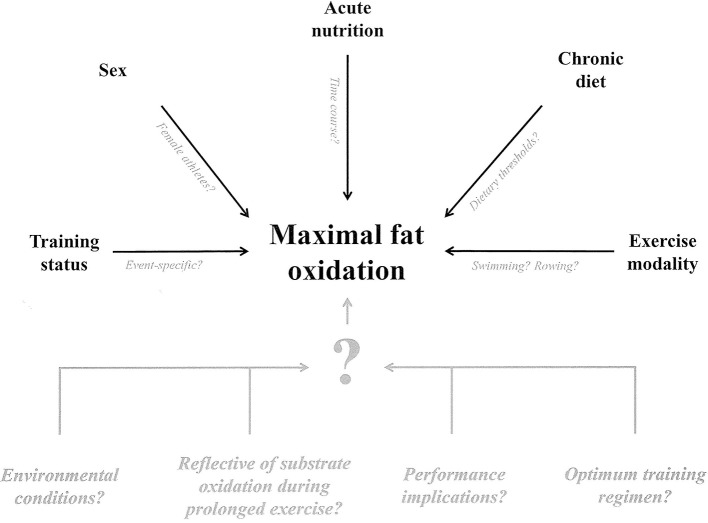
Schematic illustration of the identified determinants of maximal fat oxidation during graded protocols (black) and key identified unknown factors (gray).

Many determinants of MFO and Fat_max_ have been identified in the ~16 years since the original protocol was developed (Achten et al., 2002). However, given the practical utility of this protocol as a training monitoring tool in elite sport and as an indication of health status, further research is warranted to better understand what factors must be considered when measuring MFO and Fat_max_, as is research concerned with training effects on these variables and their relevance to endurance performance (Figure 2).

### Environmental temperature

An unexplored parameter likely to alter MFO and Fat_max_ is environmental temperature. Environmental heat stress increases muscle glycogenolysis, hepatic glucose output, and whole-body carbohydrate oxidation rates, whilst reducing fat oxidation rates at given intensities (Febbraio et al., 1994a,b; Hargreaves et al., 1996a). This is attributed to independent effects of rising core temperature, enhanced muscle temperature, greater plasma catecholamine concentrations, and progressive dehydration (Febbraio et al., 1996, 1998; Hargreaves et al., 1996b; Starkie et al., 1999). Given these effects, it might be hypothesized that MFO decreases in the heat compared to temperate conditions, although it is also possible that MFO is shifted to a lower Fat_max_. Elucidating this effect is a relevant consideration for endurance sport and military contexts given the likely negative effects of environmental heat on self-selected work intensity.

The effect of cold environments on substrate metabolism during prolonged exercise is less certain. Some investigations have reported augmented carbohydrate utilization in cold vs. temperate conditions (Galloway and Maughan, 1997; Layden et al., 2002), whereas others suggest fat utilization is augmented and carbohydrate utilization is suppressed in the cold (Galloway and Maughan, 1997; Parkin et al., 1999; Gagnon et al., 2013). Interestingly, Galloway and Maughan (Galloway and Maughan, 1997) reported greater fat oxidation rates during moderate intensity cycling at 11 vs. 21°C, but this was suppressed at 4°C. These disparities are not easily reconciled, and may be a result of interactions between the specific environmental conditions and exercise modality (cycling vs. running) (Gagnon et al., 2013).

Direct investigation of the impact of environmental temperature on laboratory measures of MFO and Fat_max_, and the environmental thresholds at which they occur, is therefore warranted. This data would have strong applied relevance given the diverse environmental conditions in which endurance competitions take place (Racinais et al., 2015; Casadio et al., 2017), as well as the extreme environments encountered in military settings (Orr et al., 2015).

### Training effects

Fourteen longitudinal studies have measured the effect of exercise training interventions on MFO and/or Fat_max_ (Venables and Jeukendrup, 2008; Mogensen et al., 2009; Alkahtani et al., 2013; Astorino et al., 2013, 2017; Besnier et al., 2015; Ipavec-Levasseur et al., 2015; Lanzi et al., 2015; Nordby et al., 2015; Rosenkilde et al., 2015; Bagley et al., 2016; Mora-Rodríguez et al., 2016; Tan et al., 2016; Schubert et al., 2017). MFO is generally upregulated in response to exercise training (Mogensen et al., 2009; Alkahtani et al., 2013; Astorino et al., 2013; Besnier et al., 2015; Ipavec-Levasseur et al., 2015; Lanzi et al., 2015; Nordby et al., 2015; Rosenkilde et al., 2015; Bagley et al., 2016; Mora-Rodríguez et al., 2016; Tan et al., 2016) whilst Fat_max_ typically remains unchanged (Venables and Jeukendrup, 2008; Mogensen et al., 2009; Alkahtani et al., 2013; Ipavec-Levasseur et al., 2015; Rosenkilde et al., 2015; Bagley et al., 2016; Astorino et al., 2017; Schubert et al., 2017), although increased Fat_max_ has been observed on occasion (Mogensen et al., 2009; Lanzi et al., 2015; Nordby et al., 2015). Training-induced increases in MFO have been consistently observed in sedentary populations (Mogensen et al., 2009; Alkahtani et al., 2013; Astorino et al., 2013; Besnier et al., 2015; Ipavec-Levasseur et al., 2015; Lanzi et al., 2015; Nordby et al., 2015; Rosenkilde et al., 2015; Mora-Rodríguez et al., 2016; Tan et al., 2016), but this effect has not always been observed in previously-active populations (Astorino and Schubert, 2017; Schubert et al., 2017), and remains uninvestigated in endurance-trained athletes.

Training-induced increases in MFO have been observed with interval (~10–80%) (Alkahtani et al., 2013; Astorino et al., 2013; Lanzi et al., 2015; Bagley et al., 2016) and moderate-intensity (~7–58%) (Venables and Jeukendrup, 2008; Mogensen et al., 2009; Alkahtani et al., 2013; Besnier et al., 2015; Ipavec-Levasseur et al., 2015; Lanzi et al., 2015; Nordby et al., 2015; Rosenkilde et al., 2015; Mora-Rodríguez et al., 2016; Tan et al., 2016) training regimens, and these responses are independent of changes in body mass (Nordby et al., 2015). Therefore, the existing literature suggests MFO is a malleable parameter that can be increased by both aerobic or interval training, particularly in sedentary populations. It is likely that training-induced increases in MFO are mediated by adaptations to adipose tissue lipolysis, NEFA transport to skeletal muscle, skeletal muscle NEFA uptake, muscle triglyceride lipolysis, and/or mitochondrial uptake of fatty acids, given fat oxidation may be limited by fatty acid delivery to skeletal muscle or mitochondrial fatty acid uptake (Romijn et al., 1993, 1995; Sidossis et al., 1997; Starritt et al., 2000; van Loon et al., 2001; Spriet, 2014). Indeed, alongside long-standing observations of adaptations to fat metabolism in response to moderate-intensity training (Howald et al., 1985; Talanian et al., 2010; Egan et al., 2011), various high-intensity or sprint interval training regimens can also stimulate beneficial adaptations across many steps involved in fat oxidation (Astorino and Schubert, 2017), including increased mitochondrial enzyme activity and protein content (Burgomaster et al., 2005, 2006, 2007, 2008; Gibala et al., 2006), muscle membrane fatty acid transport protein content (Talanian et al., 2007, 2010; Perry et al., 2008), and lipolytic enzyme protein content (Talanian et al., 2010).

The most favorable training regimen for increasing MFO cannot presently be discerned. Training studies have generally utilized either prolonged moderate-intensity aerobic exercise (Mogensen et al., 2009; Besnier et al., 2015; Ipavec-Levasseur et al., 2015; Nordby et al., 2015; Rosenkilde et al., 2015; Mora-Rodríguez et al., 2016; Tan et al., 2016) or high-intensity interval exercise (Bagley et al., 2016; Astorino et al., 2017; Schubert et al., 2017), with only three studies comparing the two (Venables and Jeukendrup, 2008; Alkahtani et al., 2013; Lanzi et al., 2015). Interestingly, differences in the magnitude of training-induced increases in MFO were not observed for moderate and high-intensity interval training in these studies (Venables and Jeukendrup, 2008; Alkahtani et al., 2013; Lanzi et al., 2015). Furthermore, whilst promising effects of training with low-glycogen availability on whole-body fat oxidation rates during prolonged exercise have been observed (Yeo et al., 2008; Hulston et al., 2010), the influence of this training regimen on MFO and Fat_max_ remains experimentally unexplored.

There is also a notable absence of data concerning the responsiveness of MFO and Fat_max_ to training in endurance-trained cohorts. Existing studies have generally been in overweight/obese populations (Venables and Jeukendrup, 2008; Mogensen et al., 2009; Alkahtani et al., 2013; Besnier et al., 2015; Ipavec-Levasseur et al., 2015; Lanzi et al., 2015; Nordby et al., 2015; Rosenkilde et al., 2015; Mora-Rodríguez et al., 2016; Tan et al., 2016), with three studies in apparently active but untrained individuals (Bagley et al., 2016; Astorino et al., 2017; Schubert et al., 2017). As endurance-trained individuals already have elevated MFO compared to lesser-trained populations, it remains to be determined if these individuals can accrue further advances in MFO through optimized training practices. It would also be useful to discern if training-induced changes in MFO reflect alterations in substrate metabolism during prolonged exercise, as the relatively short-duration of this protocol makes it a viable monitoring tool in elite sport.

Therefore, whilst it has been demonstrated that exercise training *per se* improves MFO in untrained populations, this effect remains to be elucidated in trained populations, and the most appropriate training regimen for increasing MFO is unknown. These are worthy directions for future research given the likely importance of fat oxidation capacity in endurance sport and military settings, and the apparent relationship between MFO and insulin sensitivity (Robinson et al., 2015).

### Relevance to exercise performance

A hypothesis linking MFO, Fat_max_, and performance in prolonged exercise where carbohydrate availability is limiting (>2 h) has clear intuitive appeal. If an individual makes extensive use of fat oxidation to support metabolism during prolonged exercise at their competitive or operational intensity, this should reduce the requirement for endogenous carbohydrate oxidation, and therefore muscle glycogen depletion, which is linked to fatigue (Bergström et al., 1967; Ørtenblad et al., 2013). Indeed, at a given absolute workload, significantly higher whole-body fat oxidation and lower muscle glycogenolysis have been observed in trained compared to untrained males (van Loon et al., 1999). A link between MFO, Fat_max_, and endurance exercise performance is further supported by cross-sectional evidence demonstrating enhanced MFO in trained compared to untrained cohorts (Nordby et al., 2006; Stisen et al., 2006; Ipavec-Levasseur et al., 2015).

However, the importance of MFO and Fat_max_ for exercise performance has not yet been comprehensively studied, and such research is warranted. A recent study of 64 Ironman triathletes reported a significant, albeit modest, correlation between MFO and performance time in the 2016 Copenhagen Ironman (*r* = 0.35, *P* < 0.01) (Frandsen et al., 2017). Metabolically, a cross-sectional study of elite ultra-distance runners demonstrated greater MFO and Fat_max_ in those adapted to ketogenic diets, but the rate of glycogenolysis in working skeletal muscle during prolonged exercise was not significantly different compared to those ingesting a high-carbohydrate diet, despite higher whole-body fat oxidation rates (Volek et al., 2016). Therefore, MFO, Fat_max_, and whole-body fat oxidation rates were dissociated from skeletal muscle glycogenolysis during prolonged endurance exercise between these groups, which might question the hypothesis linking MFO and Fat_max_ to endurance exercise performance via muscle glycogen sparing. However, it is possible this dissociation was an artifact of the measurement site, and that a carbohydrate sparing effect in the ketogenic group was observed in the liver, as observed previously (Webster et al., 2016).

An interesting avenue for future research might therefore be to determine if MFO and Fat_max_ are indicators of the degree of endogenous carbohydrate utilization and skeletal muscle glycogenolysis during prolonged exercise within a homogenous group of endurance-trained athletes, and consequently if such an effect has implications for endurance exercise performance. Such data would provide indication of the functional relevance of monitoring MFO and Fat_max_ in endurance-trained athletes, and could serve to build on existing models of endurance exercise performance (McLaughlin et al., 2010).

## Conclusions

This review has systematically identified several key determinants of MFO and Fat_max_. These include training status, sex, acute nutritional status, and chronic nutritional status, with the possibility of an effect of exercise modality. Accordingly, normative percentile values for MFO and Fat_max_ in different subject populations are provided to contextualize individually measured values and define the fat oxidation capacity of given research cohorts. However, the effect of environmental conditions on MFO and Fat_max_ remain to be established, as does the most appropriate means of training MFO and Fat_max_, particularly in endurance-trained cohorts. Furthermore, direct links between MFO, Fat_max_, and rates of muscle glycogenolysis during prolonged exercise remain to be established, as do relationships between MFO, Fat_max_, and exercise performance. This information might add to existing models of endurance exercise performance, and indicate how useful MFO and Fat_max_ monitoring might be in endurance sport.

## Author contributions

EM performed data analysis. EM, DP, and AK wrote the manuscript.

### Conflict of interest statement

The authors declare that the research was conducted in the absence of any commercial or financial relationships that could be construed as a potential conflict of interest.
